# Impact of the COVID-19 outbreak on the profession and psychological wellbeing of radiologists: a nationwide online survey

**DOI:** 10.1186/s13244-021-00962-2

**Published:** 2021-02-17

**Authors:** Francesca Coppola, Lorenzo Faggioni, Emanuele Neri, Roberto Grassi, Vittorio Miele

**Affiliations:** 1 Department of Radiology, IRCCS Azienda Ospedaliero-Universitaria di Bologna, Bologna, Italy; 2 Department of Translational Research, University of Pisa, Via Roma, 67, 56126 Pisa, Italy; 3 SIRM Foundation, Italian Society of Medical and Interventional Radiology, Milan, Italy; 4 Department of Precision Medicine, University of Campania, Naples, Italy; 5 Department of Radiology, Azienda Ospedaliero-Universitaria Careggi, Florence, Italy

**Keywords:** COVID-19, Radiology, Workload, Psychological disorders, Online survey

## Abstract

**Background:**

The COVID-19 outbreak has played havoc within healthcare systems, with radiology sharing a substantial burden. Our purpose is to report findings from a survey on the crisis impact among members of the Italian Society of Medical and Interventional Radiology (SIRM).

**Methods:**

All members were invited to a 42-question online survey about the impact of the COVID-19 outbreak on personal and family life, professional activity, socioeconomic and psychological condition. Participants were classified based on working in the most severely affected Italian regions (“hot regions”) or elsewhere.

**Results:**

A total of 2150 radiologists joined the survey. More than 60% of respondents estimated a workload reduction greater than 50%, with a higher prevalence among private workers in hot regions (72.7% vs 66.5% elsewhere, *p* = 0.1010). Most respondents were concerned that the COVID-19 outbreak could impact the management of non-COVID-19 patients and expected a work overload after the crisis. More than 40% were moderately or severely worried that their professional activity could be damaged, and most residents believed that their training had been affected. More than 50% of respondents had increased emotional stress at work, including moderate or severe symptoms due to sleep disturbances, feeling like living in slow motion and having negative thoughts, those latter being more likely in single-living respondents from hot regions [log OR 0.7108 (CI95% 0.3445 ÷ 1.0770), *p* = 0.0001].

**Conclusions:**

The COVID-19 outbreak has had a sensible impact on the working and personal life of SIRM members, with more specific criticalities in hot regions. Our findings could aid preserving the radiologists’ wellbeing after the crisis.

## Key points

- The COVID-19 crisis was estimated to reduce radiologists' workload by up to > 50%.
- The crisis was deemed to impact management of non-COVID-19 patients.
- Surveyed residents believed that their training had been affected.
- More than 50% of respondents experienced psychological symptoms.

## Introduction

In January 2020, a novel coronavirus strain called Severe Acute Respiratory Syndrome Coronavirus 2 was identified as the causative agent of several cases of interstitial pneumonia referred to as Coronavirus 2019 disease (COVID-19), which made its first appearance in Wuhan, China in December 2019 and has spread worldwide since then. As known, a rapidly growing number of individuals have been infected worldwide, and this unprecedented situation has put an overwhelming pressure on healthcare systems, resulting into disruption of regular workflow and potentially worse non-COVID-19 outcomes due to late or missing diagnosis and/or treatment [1–3].

Among medical specialties, radiology has shared a substantial burden due to its pivotal role in managing COVID-19. The sudden need to divert substantial resources to face the crisis has generated further issues, including lower imaging volumes, delayed procedures for non-COVID-19 patients, and curtailed educational and research activities [4–7]. Major radiological societies have striven to support radiologists by endorsing specific activities, including the production of guidelines based on available evidence and encouraging multicentric data collection and sharing [8–10].

Our purpose is to report and analyze the results of an online survey aimed to assess the impact of the COVID-19 outbreak on the working and personal life of members of the Italian Society of Medical and Interventional Radiology (SIRM).

## Methods and materials

This survey was launched by the Imaging Informatics Chapter of SIRM as a SIRM initiative to assess the impact of the COVID-19 outbreak among its members. The survey was conducted using similar methods to previous SIRM surveys [11–14].

Based upon suggestions from a multidisciplinary panel, two radiologists of the Imaging Informatics Chapter of SIRM (F.C., L.F.) created the survey using the SurveyMonkey platform (www.surveymonkey.com). The survey consisted of 42 questions (of which 36 were single choice and 6 multiple choice), grouped into five sections about demographic information and impact of the COVID-19 crisis on personal and family life, professional activity, socioeconomic condition and psychological status (Additional file 1). Each member received a personal email invitation to join the survey, which could be accessed only once over a period of one week.

Data were analyzed using SurveyMonkey Statistical Tool and a statistical software package (GraphPad Prism v. 5, www.graphpad.com). The association between survey replies expressed as categorical variables in the four regions most severely hit by the COVID-19 outbreak [15] (henceforth named “hot regions”) *versus* other regions was evaluated using the Chi-square test. Differences in the distributions of replies pertaining to emotional and interpersonal issues (i.e. Questions #9, 10, 20, 22, 23 and from 31 to 42) between male and female respondents in hot *versus* other regions, and between single-living and other respondents in hot *versus* other regions, were assessed in terms of log-odds ratios and their 95% confidence intervals, and compared using the two-tailed z test. A *p* value less than 0.05 indicates statistical significance.

## Results

### Demographic information

All survey replies are reported in Table 1.

**Table 1:** Distribution of answers in hot and other regions (see Supplement 1 for full list of answers).

Question #	Answer	Rate of replies (hot regions) 951/2150 respondents (44.2%)	Rate of replies (other regions) 1199/2150 respondents (55.8%)	χ^2^ p value
1	Under 35 years old 36–65 years old Over 65 years old	178/951 (18.7%) 649/951 (68.2%) 124/951 (13.0%)	196/1199 (16.3%) 849/1199 (70.8%) 154/1199 (12.8%)	2.23 (0.3279)
2	Male Female	469/951 (49.3%) 482/951 (50.7%)	615/1199 (51.3%) 584/1199 (48.7%)	0.75 (0.3865)
3	Hot regions (i.e. Emilia-Romagna, Lombardy, Piedmont, Veneto) Other regions	951/951 (100%) 0	0 1199/1199 (100%)	-
4	Public facility Private facility accredited to the public health service Private facility not accredited to the public health service Retired private consultant	598/951 (62.9%) 248/951 (26.1%) 29/951 (3.0%) 76/951 (8.0%)	812/1199 (67.7%) 250/1199 (20.9%) 47/1199 (3.9%) 90/1199 (7.5%)	5.3 (0.0213)*
5	Resident Post-doc fellow, PhD student or outpatient specialist Research fellow Associate or full professor Staff radiologist Medical director Private consultant	102/951 (10.7%) 11/951 (1.2%) 4/951 (0.4%) 12/951 (1.3%) 496/951 (52.2%) 81/951 (8.5%) 245/951 (25.7%)	118/1199 (9.8%) 29/1199 (2.4%) 14/1199 (1.2%) 17/1199 (1.4%) 631/1199 (52.6%) 129/1199 (10.8%) 261/1199 (21.8%)	4.48 (0.0343)*
6°	Single Partnered Married I have son(s) and/or daughter(s) I have relatives other than son(s), daughter(s), partner and/or spouse	163/912 (17.9%) 166/912 (18.2%) 543/912 (59.5%) 407/912 (44.6%) 29/912 (3.2%)	190/1164 (16.3%) 215/1164 (18.5%) 700/1164 (60.1%) 552/1164 (47.4%) 45/1164 (3.9%)	0.76 (0.3833)
7°	None Diabetes Cardiovascular diseases Respiratory diseases Immunological disorders Cancer Anxiety Depression Other	634/912 (69.5%) 24/912 (2.6%) 121/912 (13.3%) 40/912 (4.4%) 6/912 (0.7%) 40/912 (4.4%) 32/912 (3.5%) 16/912 (1.7%) 82/912 (9.0%)	827/1164 (71.0%) 26/1164 (2.2%) 166/1164 (14.3%) 43/1164 (3.7%) 9/1164 (0.8%) 26/1164 (2.2%) 39/1164 (3.3%) 17/1164 (1.5%) 89/1164 (7.6%)	0.5 (0.4795)
8	Asymptomatic with negative testing Asymptomatic with positive testing Symptomatic with negative testing Symptomatic with positive testing Asymptomatic, no testing performed Symptomatic, no testing performed	446/912 (48.9%) 10/912 (1.1%) 22/912 (2.4%) 10/912 (1.1%) 408/912 (44.7%) 16/912 (1.8%)	622/1164 (53.5%) 4/1164 (0.3%) 8/1164 (0.7%) 7/1164 (0.6%) 518/1164 (44.5%) 5/1164 (0.4%)	19.18 (< 0.0001)*
9	Yes No	693/912 (76.0%) 219/912 (24.0%)	853/1164 (73.3%) 311/1164 (26.7%)	1.83 (0.1761)
10	No Mildly Moderately Severely	107/912 (11.7%) 206/912 (22.6%) 326/912 (35.8%) 273/912 (29.9%)	123/1164 (10.6%) 299/1164 (25.7%) 404/1164 (34.7%) 338/1164 (29.0%)	0.75 (0.3865)
11	Less than 35 h 36–50 h 51–65 h More than 65 h	108/865 (12.5%) 613/865 (70.9%) 128/865 (14.8%) 16/865 (1.8%)	158/1107 (14.3%) 789/1107 (71.3%) 122/1107 (11.0%) 38/1107 (3.4%)	1.18 (0.2774)
12	Less than 35 h 36–50 h 51–65 h More than 65 h	351/865 (40.6%) 435/865 (50.3%) 71/865 (8.2%) 8/865 (0.9%)	479/1107 (43.3%) 558/1107 (50.4%) 59/1107 (5.3%) 11/1107 (1.0%)	1.34 (0.2470)
13	0–25% 25–50% 50–75% 75–100%	419/865 (48.4%) 204/865 (23.6%) 155/865 (17.9%) 87/865 (10.1%)	689/1107 (62.2%) 201/1107 (18.2%) 135/1107 (12.2%) 82/1107 (7.4%)	18.6 (< 0.0001)*
14°	I don't manage COVID-19 patients Diagnosis of COVID-19 Follow-up of COVID-19 patients Severe forms and complications of COVID-19 Other	196/865 (22.7%) 546/865 (63.1%) 482/865 (55.7%) 154/865 (17.8%) 94/865 (10.9%)	425/1107 (38.4%) 560/1107 (50.6%) 392/1107 (35.4%) 115/1107 (10.4%) 123/1107 (11.1%)	54.99 (< 0.0001)*
15	Never Rarely Quite often Very often	229/865 (26.5%) 345/865 (39.9%) 259/865 (29.9%) 32/865 (3.7%)	444/1107 (40.1%) 454/1107 (41.0%) 194/1107 (17.5%) 15/1107 (1.4%)	55.13 (< 0.0001)*
16°	Emergency procedures Oncologic imaging Non-oncologic imaging Urgent or non-deferrable interventional procedures Elective interventional procedures Other	625/865 (72.2%) 614/865 (71.0%) 406/865 (46.9%) 130/865 (15.0%) 30/865 (3.5%) 109/865 (12.6%)	784/1107 (70.8%) 730/1107 (65.9%) 441/1107 (39.8%) 123/1107 (11.1%) 21/1107 (1.9%) 168/1107 (15.2%)	0.42 (0.5169)
17	No Mildly Moderately Severely	23/865 (2.7%) 105/865 (12.1%) 455/865 (52.6%) 282/865 (32.6%)	39/1107 (3.5%) 128/1107 (11.6%) 592/1107 (53.5%) 348/1107 (31.4%)	0.01 (0.9203)
18	No Mildly Moderately Severely	62/865 (7.2%) 117/865 (13.5%) 350/865 (40.5%) 336/865 (38.8%)	95/1107 (8.6%) 166/1107 (15.0%) 455/1107 (41.1%) 391/1107 (35.3%)	2.17 (0.1407)
19	No Mildly Moderately Severely	487/865 (56.3%) 205/865 (23.7%) 132/865 (15.3%) 41/865 (4.7%)	647/1107 (58.4%) 208/1107 (18.8%) 180/1107 (16.3%) 72/1107 (6.5%)	2.03 (0.1542)
20	No Yes, they have improved Yes, they have worsened I hardly see my colleagues any more	497/865 (57.5%) 117/865 (13.5%) 108/865 (12.5%) 143/865 (16.5%)	648/1107 (58.5%) 115/1107 (10.4%) 146/1107 (13.2%) 198/1107 (17.9%)	0.88 (0.3482)
21	None Less than 10% 10–30% More than 30%	278/865 (32.1%) 482/865 (55.7%) 96/865 (11.1%) 9/865 (1.1%)	624/1107 (56.4%) 447/1107 (40.4%) 32/1107 (2.9%) 4/1107 (0.3%)	56.43 (< 0.0001)*
22	0–25% 25–50% 50–75% 75–100%	346/865 (40.0%) 269/865 (31.1%) 158/865 (18.3%) 92/865 (10.6%)	508/1107 (45.9%) 326/1107 (29.4%) 170/1107 (15.4%) 103/1107 (9.3%)	4.27 (0.0388)*
23	No Mildly Moderately Severely	145/865 (16.8%) 226/865 (26.1%) 345/865 (39.9%) 149/865 (17.2%)	171/1107 (15.5%) 318/1107 (28.7%) 443/1107 (40.0%) 175/1107 (15.8%)	0.28 (0.5967)
24	Yes No	569/865 (65.8%) 296/865 (34.2%)	710/1107 (64.1%) 397/1107 (35.9%)	0.51 (0.4751)
25	Yes No	806/865 (93.2%) 59/865 (6.8%)	996/1107 (90.0%) 111/1107 (10.0%)	5.94 (0.0148)*
26	0–25% 25–50% 50–75% 75–100%	63/856 (7.4%) 270/856 (31.5%) 372/856 (43.5%) 151/856 (17.6%)	84/1095 (7.7%) 352/1095 (32.1%) 475/1095 (43.4%) 184/1095 (16.8%)	0.13 (0.7184)
27	Yes No Don’t know	80/856 (9.3%) 425/856 (49.7%) 351/856 (41.0%)	96/1095 (8.8%) 611/1095 (55.8%) 388/1095 (35.4%)	1.04 (0.3078)
28°	Hydroalcoholic gel Masks Visors, goggles and protective gowns Other No	203/856 (23.7%) 436/856 (50.9%) 442/856 (51.6%) 45/856 (5.3%) 302/856 (35.3%)	296/1095 (27.0%) 627/1095 (57.3%) 655/1095 (59.8%) 64/1095 (5.8%) 308/1095 (28.1%)	11.11 (0.0009)*
29	Yes (symptomatic) Yes (higher risk) Yes (psychiatric conditions) No	35/856 (4.1%) 16/856 (1.9%) 13/856 (1.5%) 792/856 (92.5%)	13/1095 (1.2%) 31/1095 (2.8%) 13/1095 (1.2%) 1038/1095 (94.8%)	3.88 (0.0489)*
30	No Mildly Moderately Severely	225/856 (26.3%) 269/856 (31.4%) 280/856 (32.7%) 82/856 (9.6%)	258/1095 (23.6%) 378/1095 (34.5%) 361/1095 (33.0%) 98/1095 (8.9%)	0.01 (0.9203)
31	Yes No	30/827 (3.6%) 797/827 (96.4%)	24/1070 (2.2%) 1046/1070 (97.8%)	2.75 (0.0973)
32°	Alcohol Tobacco Other None	65/827 (7.9%) 46/827 (5.6%) 22/827 (2.7%) 716/827 (86.6%)	50/1070 (4.7%) 48/1070 (4.5%) 33/1070 (3.1%) 954/1070 (89.2%)	2.71 (0.0997)
33	No Mildly Moderately Severely	336/827 (40.6%) 286/827 (34.6%) 157/827 (19.0%) 48/827 (5.8%)	456/1070 (42.6%) 352/1070 (32.9%) 210/1070 (19.6%) 52/1070 (4.9%)	0.01 (0.9203)
34	No Mildly Moderately Severely	433/827 (52.4%) 212/827 (25.6%) 153/827 (18.5%) 29/827 (3.5%)	556/1070 (52.0%) 302/1070 (28.2%) 186/1070 (17.4%) 26/1070 (2.4%)	1.23 (0.2674)
35	Never Rarely Quite often Very often	113/827 (13.7%) 449/827 (54.3%) 231/827 (27.9%) 34/827 (4.1%)	179/1070 (16.7%) 598/1070 (55.9%) 261/1070 (24.4%) 32/1070 (3.0%)	4.66 (0.0309)*
36	Never Rarely Quite often Very often	10/827 (1.2%) 399/827 (48.3%) 388/827 (46.9%) 30/827 (3.6%)	14/1070 (1.3%) 511/1070 (47.8%) 506/1070 (47.3%) 39/1070 (3.6%)	0.01 (0.9203)
37	Never Rarely Quite often Very often	210/827 (25.4%) 345/827 (41.7%) 229/827 (27.7%) 43/827 (5.2%)	280/1070 (26.2%) 411/1070 (38.4%) 332/1070 (31.0%) 47/1070 (4.4%)	1.22 (0.2694)
38	Never Rarely Quite often Very often	113/827 (13.6%) 362/827 (43.8%) 320/827 (38.7%) 32/827 (3.9%)	134/1070 (12.5%) 530/1070 (49.5%) 361/1070 (33.8%) 45/1070 (4.2%)	3.96 (0.0466)*
39	More Less Nothing has changed	311/827 (37.6%) 260/827 (31.4%) 256/827 (31.0%)	397/1070 (37.1%) 354/1070 (33.1%) 319/1070 (29.8%)	0.5 (0.4795)
40	Never Rarely Quite often Very often	327/827 (39.5%) 372/827 (45.0%) 113/827 (13.7%) 15/827 (1.8%)	412/1070 (38.5%) 491/1070 (45.9%) 150/1070 (14.0%) 17/1070 (1.6%)	0 (1)
41	Never Rarely Quite often Very often	593/827 (71.7%) 192/827 (23.2%) 37/827 (4.5%) 5/827 (0.6%)	762/1070 (71.2%) 250/1070 (23.4%) 46/1070 (4.3%) 12/1070 (1.1%)	0.05 (0.8231)
42	More Less Nothing has changed	34/827 (4.1%) 524/827 (63.4%) 269/827 (32.5%)	51/1070 (4.8%) 627/1070 (58.6%) 392/1070 (36.6%)	3.29 (0.0697)

°Multiple answers allowed. *p < 0.05

A total of 2150 radiologist members of SIRM (amounting to 20.1% of members in good standing for the year 2020) joined the survey. The response rate for the demographics section of the survey was 100% (2150/2150).

**Fig. 1:**
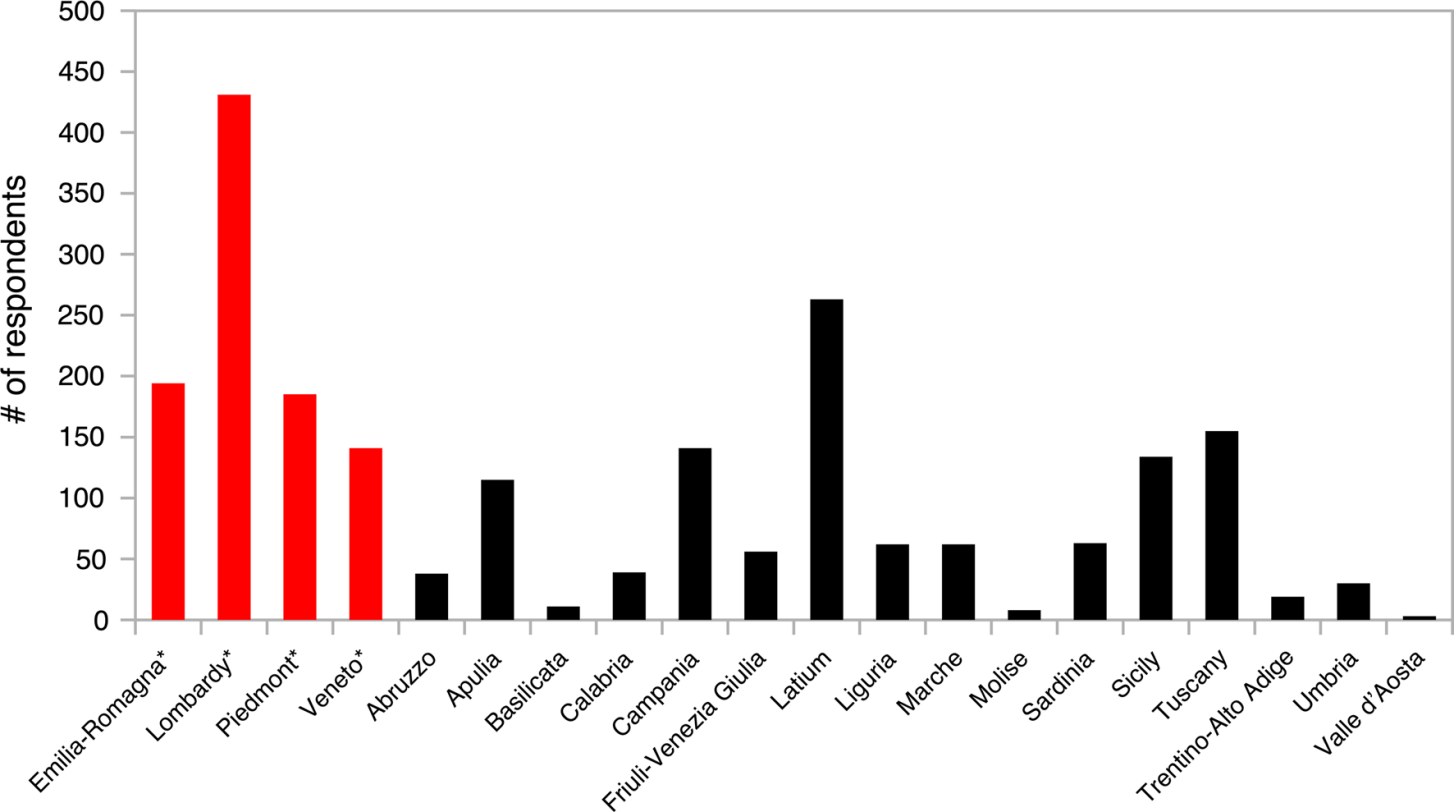
Bar chart showing the distribution of survey respondents in hot regions (asterisk, left hand side of the chart) and in other regions (right hand side)

The age (Question #1) and gender (Question #2) distributions of the survey respondents were comparable in all regions. Among respondents, 951 (44.2%) worked in hot regions and the remaining 1199 (55.8%) in other regions (Question #3) (Fig. 1). More than 60% of respondents worked at public facilities (Question #4), with a higher proportion of private workers operating in hot regions [353/951 (37.1%) vs 387/1199 (32.3%), *p* = 0.0213]. About half of respondents worked as staff radiologists (Question #5), followed by private consultants, those latter being more densely distributed in hot regions [245/951 (25.7%) vs 261/1199 (21.8%), *p* = 0.0343].

### Personal and family impact of the COVID-19 outbreak

Questions about the personal and family impact of the COVID-19 outbreak were answered by 96.6% (2076/2150) of survey participants.

More than 75% of respondents were partnered or married and slightly less than half had sons and/or daughters (Question #6). About 70% of all respondents had no known medical conditions other than possible COVID-19 (Question #7). About half of respondents were asymptomatic with negative testing (Question #8), with 44% being asymptomatic without testing. Presence of COVID-19-related symptoms was more frequent in hot regions [48/912 (5.3%) vs 20/1164 (1.7%), *p* < 0.0001].

More than 73% of respondents were afraid of passing on COVID-19 to their family members (Question #9), and about two thirds had their family relationships moderately or severely affected by the crisis (Question #10).

### Professional impact of the COVID-19 outbreak

The response rate for the professional section of the survey was 91.7% (1972/2150). More than 85% of respondents worked more than 35 h a week (including guard and on-call shifts; Question #11) before the crisis, and this proportion fell to less than 60% since that (Question #12).

In hot regions, most (i.e. > 50%) of the respondents’ professional activity was dedicated to COVID-19 (Question #13) in 242/865 cases (28.0%), more than in other regions [217/1107 cases (19.6%), *p* < 0.0001]. The proportion of respondents who did not manage COVID-19 patients (Question #14) was lower in hot regions [196/865 (22.7%) vs 425/1107 (38.4%), *p* < 0.0001], and more procedures for severe forms and complications of COVID-19 were performed in hot regions. Incidental diagnoses of COVID-19 (Question #15) were made more frequently in hot regions [291/865 (33.6%) vs 209/1107 (18.9%), *p* < 0.0001].

The most frequent procedures performed on non-COVID-19 patients during the crisis (Question #16) included emergency imaging (around 70%), followed by oncologic imaging (about two thirds of respondents). About 85% of respondents thought that the crisis could have a moderately or severely negative impact on the management of non-COVID-19 patients (Question #17), and more than 75% were moderately or severely concerned that a work overload will occur after the crisis to catch up on deferred procedures (Question #18).

Only about 20% respondents believed that the crisis had moderately or severely affected their radiological training and/or skills (Question #19), but this proportion was much higher among residents [58/86 (67.4%) from hot regions and 82/109 (75.2%) from other regions; *p* = 0.2987]. The majority of respondents denied any relevant impact of the crisis on their relationships with colleagues (Question #20). The respondents’ colleagues in hot regions had been infected more frequently than in other regions [Question #21; 105/865 (12.1%) vs 36/1107 (3.3%) of more than 10% of infected colleagues, *p* < 0.0001]. Respondents were more afraid of getting infected at work [Question #22; 250/865 (28.9%) vs 273/1107 (24.7%) over a 50% threshold, *p* = 0.0388], with male respondents from hot regions being more afraid compared to those from other regions [log OR 0.4049 (CI95% 0.1071 ÷ 0.7027), *p*= 0.0077] (Table 2).

**Table 2:** Distribution of answers in hot and other regions stratified by respondents’ gender.

Question #	% of replies (hot regions)	% of replies (other regions)	log OR (CI95%)	z (p value)
9	Male: 314/452 (69.5%) Female: 379/460 (82.4%)	Male: 399/598 (66.7%) Female: 454/566 (80.2%)	Male: 0.1265 (− 0.1860 ÷ 0.4389) Female: 0.1435 (− 0.1243 ÷ 0.4112)	Male: 0.7934 (0.4275) Female: 1.0503 (0.2936)
10	Male: 270/452 (59.7%) Female: 329/460 (71.5%)	Male: 321/598 (53.7%) Female: 421/566 (74.4%)	Male: 0.2470 (− 0.0293 ÷ 0.5233) Female: − 0.1450 (− 0.3929 ÷ 0.1029)	Male: 1.7521 (0.0798) Female: − 1.1467 (0.2515)
20	Male: 116/429 (27.0%) Female: 135/436 (31.0%)	Male: 149/569 (26.2%) Female: 195/538 (36.2%)	Male: 0.0437 (− 0.2506 ÷ 0.3380) Female: − 0.2371 (− 0.4937 ÷ 0.0195)	Male: 0.2910 (0.7710) Female: − 1.8113 (0.0701)
22	Male: 102/429 (23.8%) Female: 148/436 (33.9%)	Male: 98/569 (17.2%) Female: 175/538 (32.5%)	Male: 0.4049 (0.1071 ÷ 0.7027) Female: 0.0639 (− 0.2188 ÷ 0.3465)	Male: 2.6646 (0.0077)* Female: 0.4429 (0.6578)
23	Male: 200/429 (46.6%) Female: 294/436 (67.4%)	Male: 270/569 (47.5%) Female: 348/538 (64.7%)	Male: − 0.0334 (− 0.3093 ÷ 0.2425) Female: 0.1226 (− 0.1190 ÷ 0.3641)	Male: − 0.2372 (0.8125) Female: 0.9947 (0.3199)
31	Male: 11/406 (2.7%) Female: 19/421 (4.5%)	Male: 11/549 (2.0%) Female: 13/521 (2.5%)	Male: 0.3090 (− 0.4465 ÷ 1.0644) Female: 0.6135 (− 0.1985 ÷ 1.4256)	Male: 0.8016 (0.4228) Female: 1.4808 (0.1387)
32	Male: 59/406 (14.5%) Female: 52/421 (12.4%)	Male: 61/549 (11.1%) Female: 55/521 (10.6%)	Male: 0.3077 (− 0.0929 ÷ 0.7082) Female: 0.1773 (− 0.2086 ÷ 0.5632)	Male: 1.5053 (0.1322) Female: 0.9004 (0.3679)
33	Male: 80/406 (19.7%) Female: 125/421 (29.7%)	Male: 109/549 (19.9%) Female: 153/521 (29.4%)	Male: − 0.0094 (− 0.3312 ÷ 0.3123) Female: 0.0156 (− 0.2664 ÷ 0.2976)	Male: − 0.0575 (0.9541) Female: 0.1084 (0.9137)
34	Male: 74/406 (18.2%) Female: 108/421 (25.7%)	Male: 96/549 (17.5%) Female: 116/521 (22.3%)	Male: 0.0505 (− 0.2832 ÷ 0.3841) Female: 0.1862 (− 0.1156 ÷ 0.4881)	Male: 0.2965 (0.7668) Female: 1.2093 (0.2265)
35	Male: 102/406 (25.1%) Female: 163/421 (38.7%)	Male: 120/549 (21.9%) Female: 173/521 (33.2%)	Male: 0.1819 (− 0.1160 ÷ 0.4798) Female: 0.2397 (− 0.0327 ÷ 0.5121)	Male: 1.1968 (0.2314) Female: 1.7246 (0.0846)
36	Male: 209/406 (51.5%) Female: 209/421 (49.6%)	Male: 285/549 (51.9%) Female: 260/521 (49.9%)	Male: − 0.0174 (− 0.2901 ÷ 0.2553) Female: − 0.0104 (− 0.2503 ÷ 0.2294)	Male: − 0.1251 (0.9004) Female: − 0.0851 (0.9322)
37	Male: 116/406 (28.6%) Female: 156/421 (37.1%)	Male: 150/549 (27.3%) Female: 229/521 (44.0%)	Male: 0.0620 (− 0.2303 ÷ 0.3544) Female: − 0.2868 (− 0.5421 ÷ − 0.0316)	Male: 0.4159 (0.6775) Female: − 2.2023 (0.0276)*
38	Male: 137/406 (33.7%) Female: 215/421 (51.1%)	Male: 160/549 (29.1%) Female: 246/521 (47.2%)	Male: 0.2137 (− 0.0671 ÷ 0.4945) Female: 0.1542 (− 0.0977 ÷ 0.4061)	Male: 1.4916 (0.1358) Female: 1.1997 (0.2303)
39	Male: 103/406 (25.4%) Female: 157/421 (37.3%)	Male: 156/549 (28.4%) Female: 198/521 (38.0%)	Male: − 0.1551 (− 0.4534 ÷ 0.1433) Female: − 0.0303 (− 0.2866 ÷ 0.2260)	Male: − 1.0187 (0.3083) Female: − 0.2318 (0.8167)
40	Male: 41/406 (10.1%) Female: 87/421 (20.7%)	Male: 55/549 (10.0%) Female: 112/521 (21.5%)	Male: 0.0089 (− 0.3910 ÷ 0.4087) Female: − 0.0500 (− 0.3983 ÷ 0.2983)	Male: 0.0435 (0.9653) Female: − 0.2815 (0.7783)
41	Male: 14/406 (3.4%) Female: 28/421 (6.7%)	Male: 17/549 (3.1%) Female: 41/521 (7.9%)	Male: 0.1112 (− 0.5454 ÷ 0.7679) Female: − 0.1814 (− 0.7601 ÷ 0.3973)	Male: 0.3320 (0.7399) Female: − 0.6143 (0.5390)
42	Male: 88/406 (21.7%) Female: 181/421 (43.0%)	Male: 142/549 (25.9%) Female: 250/521 (48.0%)	Male: − 0.2317 (− 0.5366 ÷ 0.0732) Female: − 0.2015 (− 0.4585 ÷ 0.0555)	Male: − 1.4896 (0.1363) Female: − 1.5368 (0.1243)

*p < 0.05

More than 55% of respondents had their emotional stress at work moderately or severely increased (Question #23). Most respondents also felt that their professional contribution could be relevant (Question #24), and that workplaces will need to be updated after the crisis [Question #25; 806/865 (93.2%) in hot regions vs 996/1107 (90.0%) in other regions, *p* = 0.0148].

### Socioeconomic impact of the COVID-19 outbreak

The response rate for the socioeconomic section of the survey was 90.7% (1951/2150). More than 60% of respondents estimated that the crisis led to a workload reduction higher than 50% (Question #26). This figure was higher for private than for public workers [224/308 (72.7%) in hot regions vs 226/340 (66.5%) in other regions; *p* = 0.1010].

Less than 10% of respondents thought that the private sector had been adequately protected during the crisis (Question #27). The majority of respondents had difficulty obtaining personal protective equipment (PPE) (Question #28), and this percentage was lower in hot regions (64.7% vs 71.9%, *p* = 0.0009).

More than 90% of respondents were not forced to stay away from work (Question #29), but the percentage of those who did was higher in hot regions [64/856 (7.5%) vs 57/1095 (5.2%), *p* = 0.0489]. More than 40% of respondents were moderately or severely worried that the crisis will have a detrimental impact on their and their colleagues’ professional activity (Question #30).

### Psychological impact of the COVID-19 outbreak

The response rate for the psychological section of the survey was 88.2% (1897/2150). The majority of respondents did not start any psychiatric treatment (Question #31), and the use of recreational substances did not change during the crisis (Question #32).

About 40% of radiologists denied suffering from sleep disturbances during the crisis (Question #33), with moderate or severe disturbances being reported by about one quarter of respondents. Sleep disturbances mostly had a mild or no impact on daily functioning (Question #34), although sleep was moderately or severely impaired in about 20% of respondents.

A consistent minority of respondents had negative thoughts quite often or very often during the crisis (Question #35), and more so in hot regions [265/827 (32.0%) vs 293/1070 (27.4%), *p* = 0.0309]. Such likelihood was also increased in single-living respondents from hot regions than from other regions [log OR = 0.7108 (CI95% 0.3445 ÷ 1.0770), *p* = 0.0001] (Table 3).

**Table 3:** Distribution of answers in hot and other regions stratified by respondents’ living condition (i.e. single or not).

Question #	% of replies (hot regions)	% of replies (other regions)	log OR (CI95%)	z (p value)
9	Single: 115/163 (70.6%) Others: 578/749 (77.2%)	Single: 141/190 (74.2%) Others: 712/974 (73.1%)	Single: − 0.1832 (− 0.5608 ÷ 0.1944) Others: 0.2182 (− 0.1364 ÷ 0.5727)	Single: − 0.9511 (0.3415) Others: 1.2061 (0.2278)
10	Single: 124/163 (76.1%) Others: 475/749 (63.4%)	Single: 143/190 (75.3%) Others: 599/974 (61.5%)	Single: 0.0440 (− 0.3453 ÷ 0.4334) Others: 0.0819 (− 0.2721 ÷ 0.4358)	Single: 0.2216 (0.8246) Others: 0.4533 (0.6503)
20	Single: 56/155 (36.1%) Others: 195/710 (27.5%)	Single: 54/182 (29.7%) Others: 290/925 (31.4%)	Single: 0.2933 (− 0.0736 ÷ 0.6601) Others: − 0.1874 (− 0.5345 ÷ 0.1596)	Single: 1.5670 (0.1171) Others: − 1.0585 (0.2898)
22	Single: 35/155 (22.6%) Others: 215/710 (30.3%)	Single: 36/182 (19.8%) Others: 237/925 (25.6%)	Single: 0.1679 (− 0.2412 ÷ 0.5771) Others: 0.2318 (− 0.1617 ÷ 0.6253)	Single: 0.8045 (0.4211) Others: 1.1547 (0.2482)
23	Single: 83/155 (53.5%) Others: 411/710 (57.9%)	Single: 93/182 (51.1%) Others: 525/925 (56.8%)	Single: 0.0982 (− 0.2508 ÷ 0.4473) Others: 0.0462 (− 0.2722 ÷ 0.3646)	Single: 0.5515 (0.5813) Others: 0.2845 (0.7760)
31	Single: 4/150 (2.7%) Others: 26/677 (3.8%)	Single: 3/178 (1.7%) Others: 21/892 (2.4%)	Single: 0.4689 (− 0.5990 ÷ 1.5367) Others: 0.5047 (− 0.7159 ÷ 1.7253)	Single: 0.8606 (0.3895) Others: 0.8105 (0.4176)
32	Single: 23/150 (15.3%) Others: 88/677 (13.0%)	Single: 25/178 (14.0%) Others: 91/892 (10.2%)	Single: 0.1029 (− 0.3946 ÷ 0.6003) Others: 0.2739 (− 0.2013 ÷ 0.7491)	Single: 0.4053 (0.6853) Others: 1.1298 (0.2586)
33	Single: 47/150 (31.3%) Others: 158/677 (23.3%)	Single: 49/178 (27.5%) Others: 213/892 (23.9%)	Single: 0.1834 (− 0.2049 ÷ 0.5717) Others: − 0.0300 (− 0.3931 ÷ 0.3332)	Single: 0.9259 (0.3545) Others: − 0.1618 (0.8715)
34	Single: 40/150 (26.7%) Others: 142/677 (21.0%)	Single: 38/178 (21.3%) Others: 174/892 (19.5%)	Single: 0.2925 (− 0.1140 ÷ 0.6989) Others: 0.0910 (− 0.3039 ÷ 0.4859)	Single: 1.4103 (0.1584) Others: 0.4515 (0.6516)
35	Single: 59/150 (39.3%) Others: 206/677 (30.4%)	Single: 43/178 (24.2%) Others: 250/892 (28.0%)	Single: 0.7108 (0.3445 ÷ 1.0770) Others: 0.1161 (− 0.2569 ÷ 0.4892)	Single: 3.8037 (0.0001)* Others: 0.6103 (0.5417)
36	Single: 79/150 (52.7%) Others: 339/677 (50.1%)	Single: 89/178 (50.0%) Others: 456/892 (51.1%)	Single: 0.1068 (− 0.2474 ÷ 0.4609) Others: − 0.0419 (− 0.3637 ÷ 0.2799)	Single: 0.5909 (0.5546) Others: − 0.2552 (0.7986)
37	Single: 52/150 (34.7%) Others: 220/677 (32.5%)	Single: 55/178 (30.9%) Others: 324/892 (36.3%)	Single: 0.1711 (− 0.2016 ÷ 0.5439) Others: − 0.1697 (− 0.5157 ÷ 0.1763)	Single: 0.8998 (0.3682) Others: − 0.9612 (0.3364)
38	Single: 62/150 (41.3%) Others: 290/677 (42.8%)	Single: 63/178 (35.4%) Others: 343/892 (38.5%)	Single: 0.2516 (− 0.1073 ÷ 0.6105) Others: 0.1818 (− 0.1537 ÷ 0.5174)	Single: 1.3741 (0.1694) Others: 1.0621 (0.2882)
39	Single: 51/150 (34.0%) Others: 209/677 (30.9%)	Single: 55/178 (30.9%) Others: 299/892 (33.5%)	Single: 0.1416 (− 0.2336 ÷ 0.5167) Others: − 0.1214 (− 0.4684 ÷ 0.2256)	Single: 0.7396 (0.4595) Others: − 0.6856 (0.4930)
40	Single: 27/150 (18.0%) Others: 101/677 (14.9%)	Single: 26/178 (14.6%) Others: 141/892 (15.8%)	Single: 0.2494 (− 0.2177 ÷ 0.7166) Others: − 0.0683 (− 0.5215 ÷ 0.3849)	Single: 1.0466 (0.2953) Others: − 0.2956 (0.7675)
41	Single: 10/150 (6.7%) Others: 32/677 (4.7%)	Single: 9/178 (5.1%) Others: 49/892 (5.5%)	Single: 0.2936 (− 0.4396 ÷ 1.0268) Others: − 0.1584 (− 0.8881 ÷ 0.5714)	Single: 0.7849 (0.4325) Others: − 0.4254 (0.6705)
42	Single: 51/150 (34.0%) Others: 218/677 (32.2%)	Single: 64/178 (36.0%) Others: 328/892 (36.8%)	Single: − 0.0860 (− 0.4603 ÷ 0.2883) Others: − 0.2025 (− 0.5375 ÷ 0.1325)	Single: − 0.4502 (0.6526) Others: − 1.1847 (0.2361)

*p < 0.05

The percentage of respondents being in a good mood was comparable to those who were not (Question #36). However, more than 30% of respondents felt quite often or very often like living in slow motion (Question #37), with females from hot regions being less susceptible than those from other regions [log OR − 0.2868 (CI95% − 0.5421 ÷ − 0.0316), *p* = 0.0276]. More respondents from hot regions than from other regions felt restless or nervous quite often or very often (Question #38) [352/827 (42.6%) vs 406/1070 (37.9%), *p* = 0.0466].

About 30% of respondents enjoyed relaxing and doing the same things as before the crisis (Question #39). A relatively small proportion of respondents complained about having quite often or very often feelings of fear (Question #40, about 15%) or panic (Question #41, about 5%). About 60% of respondents took less care of their physical appearance during the crisis (Question #42).

## Discussion

To our knowledge, this is the first survey by a European national radiological society to assess the impact of the COVID-19 pandemic on its members’ overall wellbeing.

Our survey has revealed a sensible impact of the COVID-19 outbreak on radiologists’ life, with differences in specific items occurring in hot regions. The majority of respondents were afraid of spreading the infection and felt that the crisis had affected their family relationships and will damage their own and their colleagues’ professional activity. Such concerns were independent of the severity of the crisis on a per-region basis, suggesting a generalized discomfort and emotional distress, as also highlighted by answers to the psychological part of the survey.

However, radiologists from hot regions were understandably more afraid of getting infected at work and were also more exposed than those from other regions in terms of a more intensive management of COVID-19 patients, more incidental diagnoses of COVID-19, and a higher proportion of infected colleagues. In a Chinese survey on mental health outcomes of 1257 healthcare professionals (of whom 60% operating in Wuhan), psychological distress was more common in frontline healthcare workers engaged in direct diagnosis, treatment and care of COVID-19 patients, with participants from outside Hubei province being at a lower risk of experiencing distress than those in Wuhan [16]. In another Chinese survey among radiology departments in 32 public hospitals, respondents showed an overall low toughness dimension score, along with a negative correlation between respondents’ perceived stress and resilience. This survey also showed that knowledge of COVID-19, knowledge of COVID-19 protective measures and availability of adequate protective materials were independent influencing factors for resilience [17]. Although more than half of our survey respondents had difficulty sourcing PPE, such difficulty was higher in non-hot regions, which might have been less prepared to the crisis as hot regions experienced its peak with some time advance. This should be taken into account for planning the activity of radiology departments during the post-shutdown phase of the COVID-19 pandemic, in order to preserve the mental wellbeing of frontline providers and maximize safety, especially in case of pandemic resurgence [7, 18]. Of note, the crisis apparently cemented most respondents’ belief that their professional contribution could be relevant and did not alter the relationships with colleagues, indicating that radiology teams have faced the crisis in a cohesive manner.

A decrease of working hours before and during the crisis was reported, with private radiological facilities being perceived as more severely stricken and less protected by healthcare authorities compared to the public health system. While overall imaging volumes have shrunk (with a loss up to 80% of procedures at the height of the crisis), the private sector has been especially affected due to a reduced demand from patients, shunning medical services out of fear of contracting COVID-19 and over a period of economic setback [19–22]. This is in line with our finding of a higher proportion of respondents from private than from public facilities estimating a workload reduction greater than 50%. Moreover, the fact that non-public radiology centers were commoner in hot regions could have resulted in greater damage to the healthcare system in areas more critically affected by the crisis.

A shift in workload has also been observed related to non-COVID-19 patients, with emergency procedures making up the most (about 70%) of radiology workload. This change was associated to most radiologists’ fear of having to catch up on deferred procedures and that the crisis could negatively impact the management of non-COVID-19 patients. Norbash et al. reported an overall volume drop of radiological procedures from 40 to 70% compared with the same period of the previous year, plummeting to 99% for breast screening examinations [19]. Boeken et al. reported a 61% volume drop in emergency CT examinations of non-COVID-19 patients performed some days before the COVID-19 lockdown compared to the same period of the previous year, and their finding of no difference in the distribution of positive CT scans would suggest that potentially severe conditions could go undiagnosed [22]. Still, in our survey the predominance of emergency and oncologic imaging in non-COVID-19 patients showed that radiology facilities have tried to guarantee life-critical procedures despite the crisis, in an attempt to minimize any potential major health hazard [23].

The overall impoverishment of radiological activity could have had a negative impact on the skills of training personnel, as perceived by most radiology residents. Concerns have been expressed by trainees and medical educators that the effectiveness of education programs could be jeopardized by social distancing, limited regular clinical and research activities with a reduced ability to perform live education sessions and hands-on practice, changes in residents’ rotations or postponement of core examinations [24–26]. A perceived worse resident morale in radiology residency programs with redeployment was reported in a survey by the Well-Being Subcommittee of the Association of Program Directors in Radiology (APDR), along with a moderate/marked negative impact on the educational mission (70.1% of respondents) and a mildly or markedly decreased morale of program directors (61%) [24]. Some potential solutions have been proposed, including virtual meetings for e-learning and participation in remotely accessible research opportunities, simulated daily readout sessions during protected education time slots, reconfiguring rotations to ensure distancing while enabling preparation for delayed core exams, and avoiding multiple proceduralists for interventional procedures whenever feasible [24–29].

Attention should also be paid to the impact of the COVID-19 outbreak on the psychological status of radiology workers, resulting in a variable impairment that should be counteracted by taking appropriate actions [30–33]. Although psychological disturbances were most often tolerable as to not require specific treatment, moderate or even severe manifestations of depressive and/or anxiety symptoms occurred in a very consistent minority of respondents. Moreover, respondents from hot regions were more subject to some symptoms (including having negative thoughts or feeling restless or nervous), reflecting an association between exposure to the crisis and psychological damage. In a web-based survey conducted among Chinese healthcare workers during the COVID-19 pandemic, psychological issues were commoner in frontline workers, with a prevalence of anxiety, depression, insomnia and overall psychological problems of 46.04%, 44.37%, 28.75% and 56.59%, respectively [31]. In a systematic meta-analysis including 13 studies and a combined total of 33,062 participants, the pooled prevalence of anxiety and depression was slightly higher than 20%, whereas the same figure for insomnia was about 40% [32]. While females have been reported to have a higher risk of psychological symptoms [16, 32, 33], in our survey most symptoms had no gender prevalence, except for negative thoughts being commoner in women from hot regions. Interestingly, women from hot regions were less susceptible to feeling like living in slow motion than those from other regions, possibly suggesting a greater sense of control in workplaces more directly affected by the crisis, which could be a major driver of engagement and contribute to avoid burnout [34]. Moreover, in our survey the likelihood of having negative thoughts was higher among single-living respondents from hot regions compared to the same from other regions, supporting the role of social isolation as a potential risk factor for anxiety and depression in COVID-19 workers [33].

Our study has a potential limitation in that, although the participation rate was higher compared to previous SIRM surveys [11–14], only one fifth of SIRM members joined our survey, hence our findings might not be representative of the entire SIRM population. However, such circumstance seems unlikely because unfortunately the COVID-19 outbreak has been a nationwide emergency with similar problems across the country (albeit more severe in hot regions). It is also possible that our short survey duration of one week might have limited the number of participants and prevented us from obtaining further information. However, we restricted the survey duration to avoid the risk of bias due to collecting data over an extended time frame amid a rapidly changing emergency situation.

A further limitation is the lack of one or more reference groups of survey participants other than radiologists (including physicians from other specialties, technologists, nurses, and/or a general public outside the healthcare environment), potentially allowing to better discriminate findings specific to radiologists from those (e.g. symptoms of psychological distress) that might be shared by other groups. However, this would have involved splitting our survey (which contains many radiology-specific questions) into separate, more general parts to be administered to nonradiologists as well, thereby increasing its overall complexity and carrying the risk of bias due to inhomogeneity of data collected from different populations. While, in order to be meaningful, such an extended survey should have been performed simultaneously to that we actually conducted, we are planning on devising a further survey including a nonradiologist audience as a reference, in an attempt to corroborate our findings on a larger population from a wider perspective.

## Conclusions

Our survey shows that the working and personal life of SIRM members has been impacted by the COVID-19 outbreak, with more specific patterns of involvement in hot regions due to their higher exposure. While our survey was restricted to radiologists and did not take into account other respondents’ groups as a reference, we believe that specific recommendations should be drawn up based on our findings, including the following:

- To invest on professional training of all involved personnel, striving to provide universal access to PPE while fostering appropriate use of such equipment, thus maximizing safety and avoiding PPE misuse or waste.
- To encourage a responsible use of imaging in radiologists, referring clinicians and patients by improving the adherence to established guidelines and clinical decision support systems, in order to exploit available imaging resources in the most efficient manner while minimizing unnecessary procedures (and hence, potentially avoidable exposure of patients and staff to COVID-19 infection).
- To separate COVID-19 patients’ pathways from those of other patients to the maximum possible extent, by dedicating specific environments and working shifts inside radiological departments and upgrading those latter to guarantee distancing, cleanliness and lack of contamination.
- To increase the frequency of shift rotations for the entire radiological staff (including residents), to promote team working (as much as allowable in relation to distancing requirements and personnel availability) and to provide psychological support, in order to minimize the risk of burnout and psychological discomfort. Financial support should also be considered for private workers.

## Supplementary information

**Additional file 1**. Survey form with answers in full for each question. *hot region.

## Abbreviations

COVID-19: Coronavirus 2019 disease
PPE: Personal protective equipment
