# Supplementary material for: Impact of the COVID-19 outbreak on the profession and psychological wellbeing of radiologists: a nationwide online survey
Source: Insights Imaging. 2021 Feb 17;12:23. doi: 10.1186/s13244-021-00962-2 (PMC7887566; doi:10.1186/s13244-021-00962-2)
Supplement: Supplementary file 1 — Additional file 1. Survey form with answers in full for each question. *hot region. [file 13244_2021_962_MOESM1_ESM.docx]

**PART 1. DEMOGRAPHIC INFORMATION**

1. What is your age?
   1. under 35 years old
   2. 36-65 years old
   3. over 65 years old
2. Which gender are you?
   1. male
   2. female
3. Which Italian region do you work in?
   1. Abruzzo
   2. Apulia
   3. Basilicata
   4. Calabria
   5. Campania
   6. Emilia-Romagna*
   7. Friuli-Venezia Giulia
   8. Latium
   9. Liguria
   10. Lombardy*
   11. Marche
   12. Molise
   13. Piedmont*
   14. Sardinia
   15. Sicily
   16. Tuscany
   17. Trentino-Alto Adige
   18. Umbria
   19. Valle d’Aosta
   20. Veneto*
4. Who is your employer?
   1. public facility
   2. private facility accredited to the public health service
   3. private facility not accredited to the public health service
   4. I work as a retired private consultant
5. What is your professional role?
   1. resident
   2. post-doc fellow, PhD student or outpatient specialist
   3. research fellow
   4. associate or full professor
   5. staff radiologist
   6. medical director
   7. private consultant

**PART 2. PERSONAL AND FAMILY IMPACT OF THE COVID-19 OUTBREAK**

1. What is your family status? (MULTIPLE ANSWERS)
   1. single
   2. partnered
   3. married
   4. I have son(s) and/or daughter(s)
   5. I live with relatives other than son(s), daughter(s), partner and/or spouse
2. Do you have any medical condition (other than possible COVID-19)? (MULTIPLE ANSWERS)
3. none
4. diabetes
5. cardiovascular diseases (high blood pressure, heart failure, etc.)
6. respiratory diseases (asthma, COPD, etc.)
7. immunological disorders
8. cancer
9. anxiety
10. depression
11. other
12. What is your health condition with respect to COVID-19?
    1. asymptomatic with negative testing
    2. asymptomatic with positive testing
    3. symptomatic with negative testing
    4. symptomatic with positive testing
    5. asymptomatic, no testing performed
    6. symptomatic, no testing performed
13. Are you afraid of passing on COVID-19 to your family members?
    1. yes
    2. no
14. Have your family relationships been affected by the COVID-19 outbreak?
    1. no
    2. mildly
    3. moderately
    4. severely

**PART 3. PROFESSIONAL IMPACT OF THE COVID-19 OUTBREAK**

1. How many hours a week did you work before the COVID-19 outbreak (including guard and on-call shifts)?
   1. less than 35
   2. 36-50
   3. 51-65
   4. more than 65
2. How many hours a week have you been working since the beginning of the COVID-19 outbreak (including guard and on-call shifts)?
   1. less than 35
   2. 36-50
   3. 51-65
   4. more than 65
3. What percentage of your professional activity is dedicated to COVID-19?
   1. 0-25%
   2. 25-50%
   3. 50-75%
   4. 75-100%
4. How is your professional activity organized with COVID-19 patients? (MULTIPLE ANSWERS)
   1. I don't manage COVID-19 patients
   2. I perform tests for the diagnosis of COVID-19
   3. I perform tests for the follow-up of COVID-19 patients
   4. I perform diagnostic and/or interventional procedures for the management of severe forms and complications of COVID-19
   5. other
5. Did you happen to make an incidental diagnosis of COVID-19?
   1. never
   2. rarely
   3. quite often
   4. very often
6. Since the beginning of the COVID-19 outbreak, which diagnostic and/or interventional radiology procedures have you been carrying out in non-COVID-19 patients? (MULTIPLE ANSWERS)
   1. emergency imaging
   2. oncologic imaging (including breast imaging)
   3. non-oncologic imaging
   4. urgent or non-deferrable interventional procedures (e.g. transcatheter arterial embolization for active bleeding, biopsies, etc.)
   5. elective interventional procedures (e.g. uterine fibroid embolization,

varicocele sclerotherapy, etc.)

- 1. other

1. Do you think that the COVID-19 outbreak could negatively impact the management of non-COVID-19 patients?
   1. no
   2. mildly
   3. moderately
   4. severely
2. Are you concerned that the COVID-19 outbreak will be followed by a work overload to catch up on procedures that were postponed due to the crisis?
   1. no
   2. mildly
   3. moderately
   4. severely
3. Has the COVID-19 outbreak negatively impacted your radiological training and/or skills?
   1. no
   2. mildly
   3. moderately
   4. severely
4. Have your relationships with your colleagues changed during the COVID-19 outbreak?
   1. no
   2. yes, they have improved
   3. yes, they have worsened
   4. I hardly see my colleagues any more
5. What percentage of your colleagues have contracted COVID-19 infection?
   1. none
   2. less than 10%
   3. 10-30%
   4. more than 30%
6. How afraid are you of getting infected at work?
   1. 0-25%
   2. 25-50%
   3. 50-75%
   4. 75-100%
7. Since the beginning of the COVID-19 outbreak, has your emotional stress at work increased?
   1. no
   2. mildly
   3. moderately
   4. severely
8. Do you think that you could provide any relevant professional contribution during the COVID-19 outbreak?
   1. yes
   2. no
9. Do you think that workplaces (e.g. reporting and waiting rooms) will need to be updated after the end of the COVID-19 outbreak (so-called “phase 2”)?
   1. yes
   2. no

**PART 4. SOCIO-ECONOMIC IMPACT OF THE COVID-19 OUTBREAK**

1. What do you estimate to be the workload reduction at your department or office due to the COVID-19 outbreak?
   1. 0-25%
   2. 25-50%
   3. 50-75%
   4. 75-100%
2. Do you think that the private sector has been adequately protected during the COVID-19 outbreak?
   1. yes
   2. no
   3. don’t know
3. Did you have difficulty sourcing the following pieces of personal protective equipment (PPE)? (MULTIPLE ANSWERS):
   1. hydroalcoholic gel
   2. masks
   3. visors, goggles and protective gowns
   4. other
   5. no
4. Are you (or have you been) forced to stay away from work due to the COVID-19 outbreak?
   1. yes, because I have symptoms of COVID-19
   2. yes, because I am at higher risk due to comorbidities (e.g. respiratory diseases, immunosuppression, etc.)
   3. yes, due to psychiatric conditions (e.g. anxiety, depression, etc.)
   4. no
5. Are you worried that the COVID-19 outbreak will have a detrimental impact on your and your colleagues' professional activity (e.g. due to failure to accomplish technological upgrading, project deferral or cancellation, loss of employment, etc.)?
   1. no
   2. mildly
   3. moderately
   4. severely

**PART 5. PSYCHOLOGICAL IMPACT OF THE COVID-19 OUTBREAK**

1. Since the beginning of the COVID-19 outbreak, have you started a treatment for

anxiety and/or depression or some other psychiatric treatment?

- 1. yes
  2. no

1. Since the beginning of the COVID-19 outbreak, have you increased or started the use of the following? (MULTIPLE ANSWERS)
   1. alcohol
   2. tobacco
   3. other
   4. I have not increased nor started the use of any substances
2. Since the beginning of the COVID-19 outbreak, have you suffered from sleep disturbances (e.g. having difficulty falling asleep or staying asleep? waking up too early in the morning)?
   1. no
   2. mildly
   3. moderately
   4. severely
3. If you have suffered from sleep disturbances since the beginning of the COVID-19 outbreak, how much do you think that your daily functioning has been damaged (e.g. due to tiredness, decreased concentration and memory, mood worsening)?
   1. no
   2. mildly
   3. moderately
   4. severely
4. Since the beginning of the COVID-19 outbreak, have you ever had negative thoughts?
   1. never
   2. rarely
   3. quite often
   4. very often
5. Since the beginning of the COVID-19 outbreak, have you ever been in a good mood?
   1. never
   2. rarely
   3. quite often
   4. very often
6. Since the beginning of the COVID-19 outbreak, have you ever felt like living in slow motion?
   1. never
   2. rarely
   3. quite often
   4. very often
7. Since the beginning of the COVID-19 outbreak, have you ever felt restless or nervous?
   1. never
   2. rarely
   3. quite often
   4. very often
8. Since the beginning of the COVID-19 outbreak, have you ever enjoyed relaxing and doing the same things as before?
   1. more
   2. less
   3. nothing has changed
9. Since the beginning of the COVID-19 outbreak, have you ever had feelings of fear that something awful should happen to you?
   1. never
   2. rarely
   3. quite often
   4. very often
10. Since the beginning of the COVID-19 outbreak, have you ever experienced sudden feelings of panic?
    1. never
    2. rarely
    3. quite often
    4. very often
11. Since the beginning of the COVID-19 outbreak, how much have you taken care of your physical appearance?
    1. more than before
    2. less than before
    3. nothing has changed
